# The associated risk of *Blastocystis* infection in cancer: A case control study

**DOI:** 10.3389/fonc.2023.1115835

**Published:** 2023-02-20

**Authors:** Lena Labania, Sumaya Zoughbor, Suad Ajab, Marie Olanda, Sulaiman N. M. Shantour, Zakeya Al Rasbi

**Affiliations:** ^1^ Microbiology and Immunology, College of Medicine and Health Sciences, United Arab Emirates University, Abu Dhabi, United Arab Emirates; ^2^ Zayed Bin Sultan Center for Health Sciences, College of Medicine and Health Sciences, United Arab Emirates University, Abu Dhabi, United Arab Emirates; ^3^ Institute of Public Health, College of Medicine and Health Sciences, United Arab Emirates University, Abu Dhabi, United Arab Emirates; ^4^ General Surgery Division, Surgery Department, Tawam Hospital, Abu Dhabi, United Arab Emirates

**Keywords:** colorectal cancer, ST subtypes, phylogenetic analysis, UAE, *Blastocystis* infection, cancer, Fungi

## Abstract

**Background:**

*Blastocystis* is an anaerobic intestinal protozoan. Nine *Blastocystis* subtypes (STs) were detected in humans. A subtype-dependent association between *Blastocystis* and different cancer types has been debated in many studies. Thus, this study aims to assess the possible association between *Blastocystis* infection and cancer, especially colorectal cancer (CRC). We also screened the presence of gut fungi and their association with *Blastocystis*.

**Methods:**

We used a case-control design; cancer patients and cancer-free (CF) participants. The cancer group was further sub-group into CRC group and cancers outside the gastrointestinal tract (COGT) group. Macroscopic and microscopic examinations were performed to identify intestinal parasites in participants’ stool samples. Molecular and phylogenetic analyses were conducted to identify and subtype *Blastocystis*. Furthermore, gut fungi were investigated molecularly.

**Results:**

104 stool samples were collected and matched between CF (n=52) and cancer patients (n=52); CRC (n=15) and COGT (n=37). As anticipated, *Blastocystis* prevalence was significantly higher among CRC patients (60%, P=0.002) and insignificant in COGT patients (32.4%, *P*=0.161) compared to CF group (17.3%). The most common subtypes were ST2 among cancer group and ST3 in the CF group.

**Conclusion:**

Cancer patients have a higher risk of *Blastocystis* infection compared to CF individuals (OR=2.98, *P*=0.022). Increased risk of *Blastocystis* infection was associated with CRC patients (OR=5.66, *P*=0.009). Nevertheless, further studies are required to understand the underlying mechanisms of *Blastocystis* and cancer association.

## 1 Introduction

*Blastocystis* is an anaerobic intestinal protozoan found in humans and a wide range of animals. Morphological forms of *Blastocystis* include vacuolar, granular, amoeboid, and cyst forms, the vacuolar form is predominant in fresh stool samples and laboratory cultures (1). The *Blastocystis* prevalence rate is 60% in developing countries, due to poor hygiene and close contact with animals, compared to developed countries (5-20%) (2). More than 17 subtypes (STs) of *Blastocystis* spp. are known, and only nine subtypes are found in humans (3, 4). *Blastocystis* was considered a commensal parasite and caused asymptomatic infections at most. However, there is an increasing number of studies investigating the role and pathogenicity of *Blastocystis* in the gut (3, 4).

Some studies showed that *Blastocystis* infections contributed to the severity of multiple conditions, such as AIDs, cancer, IBD, and gut microbiota dysbiosis (5–7). Conversely, other studies have shown that the presence of *Blastocystis* promotes high diversity of gut microbiota in healthy individuals preventing intestinal disorders (8, 9). *Blastocystis* culture filtrates did not affect the growth of cancer cell lines in one study (10). These conflicting findings are probably attributed to the genetic diversity of *Blastocystis*, inter and intra-subtype variations (7, 11, 12).

Globally, cancer is regarded as one of the leading causes of death with an estimation of 10 million deaths in 2020 (13). The most common cancers in order are breast, lung, colorectal, prostate, skin, and stomach cancers according to WHO in 2020 (13). Some of risk factors include age, family history, obesity, diet, and infectious pathogens (13, 14). Since 30-50% of cancer is preventable by avoiding its risk factors and 30% of cancer are caused by infectious pathogens, it is important to identify all possible cancer-promoting infections to limit progression of existing cases and emergence of new cases (13). Colorectal cancer (CRC) is the third most common cancer, and the second leading cause of cancer-related deaths worldwide (2020) (15). CRC cases are detected in males more than females. The majority of CRC cases are diagnosed in the late stages of the disease, partially due to its non-specific symptoms (16–19). One of the risk factors of CRC is the gut microbiota dysbiosis (17–20). Several studies have investigated the role of microbiota in different cancers initiation or progression (21–27). However, previous studies focused on the bacterial content overlooking other micro-organisms such as protozoa and fungi (28).

Since *Blastocystis spp.* is considered a normal intestinal flora, investigating *Blastocystis* association with cancer, focusing on CRC, is essential to understanding the gut microbiota effect on tumor initiation and progression (8, 29). Many studies investigated the interaction between *Blastocystis* spp. and gut microbiota, and one study reported an association between *Blastocystis* with increased levels of five gut fungi (Mycobiota) (7–9, 30). Other studies associated gut mycobiota with carcinogenesis, initiation, and development of different cancers (20, 31–33). Thus, this study aimed to assess the possible association between *Blastocystis* infection and fungi in cancer patients locally. Then, compare *Blastocystis* infection in different cancers’ patients to cancer-free controls (CF).

## 2 Methods

### 2.1 Study design

In this observational study, a matched case-control study design was used. The case patients affected by cancer were gender and age-group-matched with a CF control group. The study participants were recruited from March 2020 to April 2022. This study followed the STROBE guidelines (34).

### 2.2 Study population and variables

All participants signed informed consent (n=104), and the study was performed per the Declaration of Helsinki. Participants of both genders were 18 years old and above. Age was categorized into three groups: Youth (18-24 years old), adults (25-59 years old), and elderly (≥60 years old). Nationalities were classified into six regions of origin: Africa, Americas, South-East Asia, Europe, Eastern Mediterranean, and Western Pacific. Patients who took any anti-parasitic drug in the last six months, did not provide informed consent, or were unwilling to give a stool sample were excluded from this study. The consumption of antibiotics, gastrointestinal surgery history in the past two years, hospitalization, cancer therapy, and the number of therapy cycles were extracted from patients’ medical records.

### 2.3 Case patients

Patients referred to the Oncology Services at Tawam Hospital, United Arab Emirates, with confirmed cancer (n=52) cases histopathologically, in any stage, and undergoing any treatment were included in this study. The cases were further sub-categorized into two groups: CRC (n=15) and cancers outside the gastrointestinal tract (COGT) (n=37) patients.

### 2.4 Control participants

Community-based control participants (n=52) were gender- and age-group matched subjects recruited. Excluding subjects with intestinal disorders, immunological or neoplastic disorders, and antibiotics users in the last six months before recruitment.

### 2.5 Sample collection and processing

Stool samples were collected using a commercial stool collection kit (alpha laboratories, UK). All stool samples were transported for analysis to the Microbiology Laboratory, College of Medicine and Health Sciences (CMHS), United Arab Emirates University (UAEU). Stool samples were each split into two parts; one was stored at 4°C and processed within one to two days for macroscopy, and microscopy, while the second was put in Eppendorf tubes and stored at -20°C for molecular work.

### 2.6 Macroscopic and microscopic investigation

As mentioned previously (35), macroscopic and microscopic examinations were performed to define the types of organisms and check for blood and mucus. Stool samples were stained with Wheatley Trichrome for *Blastocystis* detection, following the manufacturer’s instructions. Then, smears were examined microscopically under ×40 and ×100 magnification objectives. Two microbiologists examined all stool slides independently.

### 2.7 Molecular investigation

Genomic DNA was extracted from stool samples as previously published (35). Primers and PCR conditions used are listed in **Table S1**. *Blastocystis* spp. and gut fungi DNAs were amplified by Polymerase Chain Reaction (PCR). A mix of 5µl of 5x Q-Solution, 2.5µl of 10x CoralLoad PCR buffer, 1µl of each primer (10mM), 0.5µl of QIAGEN Taq DNA Polymerase (250U),1µl of dNTP Blend (100mM) (Applied Biosystems, USA), and 2µl of stool genomic DNA diluted in free-nuclease water to reach final volume of 25µl. For the ITS reaction mixtures, an extra 0.5µl of MgCl_2_ was added.

### 2.8 Sequencing and phylogenetic analysis

Gel and PCR Clean-Up System (Promega, Madison, Wisconsin, USA) was used following the manufacturer’s instructions. Barcode region and ITS primers were used to sequence the purified products in both directions *via* capillary electrophoresis using a Big DyeTM Terminator Cycle Sequencing Kit (Applied Biosystems, Foster City, CA, USA) in ABI PRISM 3130xI Genetic Analyzer. The confirmed *Blastocystis*-positive sequences were assigned to the best-matched *Blastocystis* subtype by aligning them to reference sequences in the GenBank database using nBLAST program (36). Query cover and per identity of ≥97% were used to determine subtypes matches. The ClustalW algorithm of MEGA-X was used to align the sequences (37). Acquired sequences were submitted to PubMLST database to confirm subtypes and identify relevant alleles (38). The established fungi-positive sequences were assigned to the best-match fungi species by aligning them to reference sequences in the GenBank database. Query cover of ≥80% and 97-100% per identity were used to determine the most probable match.

*Blastocystis* samples and reference sequences along with an outgroup species (*Proteromonas lacertae*, GenBank accession no. U37108) went through phylogenetic analysis. The best substitution models were determined *via* the Bayesian information criterion. The maximum Likelihood (ML) method and Bayesian Inference (BI) were used to construct the tree. ML tree was constructed using MEGA-X. Tamura 3-parameter with gamma distribution was used, and Bootstrapping analysis with 1000 replicates was performed. For the BI method, Jmodeltest v2.1.10 and MrBayes v3.2.7 were used (39, 40). Hasegawa-Kishino-Yano model with gamma distribution was used, and four Markov chains were run for 5 million generations, with a sampling frequency of 100 and a 25% burn-in. Tree Graph 2 combined the two constructed trees (41).

### 2.9 Statistical analysis

Data were analyzed by IBM SPSS Statistics v26.0. Qualitative variables were expressed as numbers and percentages, while quantitative variables were expressed as means and medians. The chi-square, Fisher’s Exact, and Fisher-Freeman-Halton tests for categorical variables. Logistic regression was used to predict factors associated with *Blastocystis* prevalence, and a *p*-value of ≤ 0.05 indicated statistical significance.

### 2.10 Ethical approval

The study was approved by the Tawam Human Research Ethics Committee (T-HREC) of Tawam Hospital, Al Ain, Abu Dhabi, UAE (THREC-678).

## 3 Results

In this case-control study, a total of 104 matched participants were recruited. The controls were CF participants (n=52), and the cases were cancer patients (n=52) (**Table S2**). The study participants consisted of 44 males (42.3%) and 60 females (57.7%). CF participants’ mean age was 43.3 (median: 41.5, range: 23-87). Cancer patients’ mean age was 49.3 (median: 51, range: 22-75). There was no statistical association between *Blastocystis* infection and gender (*P*=0.147) nor *Blastocystis* infection and age groups (*P*=0.277). The prevalence of *Blastocystis* among regions differed but was statistically insignificant (*P*=0.056).

Of the cancer patients, 37 patients were COGT, while 15 individuals were CRC patients. COGT patients were of 19 different cancer types reclassified into 8 categories based on tumor site (**Table S2**). The majority of patients had breast cancer (n=16, 15.4%), and hematologic cancer (n=9, 8.7%). The *Blastocystis* prevalence within cancer types was statistically insignificant (*P*=0.440). Also, antibiotics usage, GIT surgery history, number of chemotherapy cycles and hospitalization were statistically insignificant to *Blastocystis* infection (*P*=0.721, *P*=0.07, *P*=0.705, and *P*=0.241) in cancer group (**Table S3**).

### 3.1 Stool analysis

**Figure 1:**
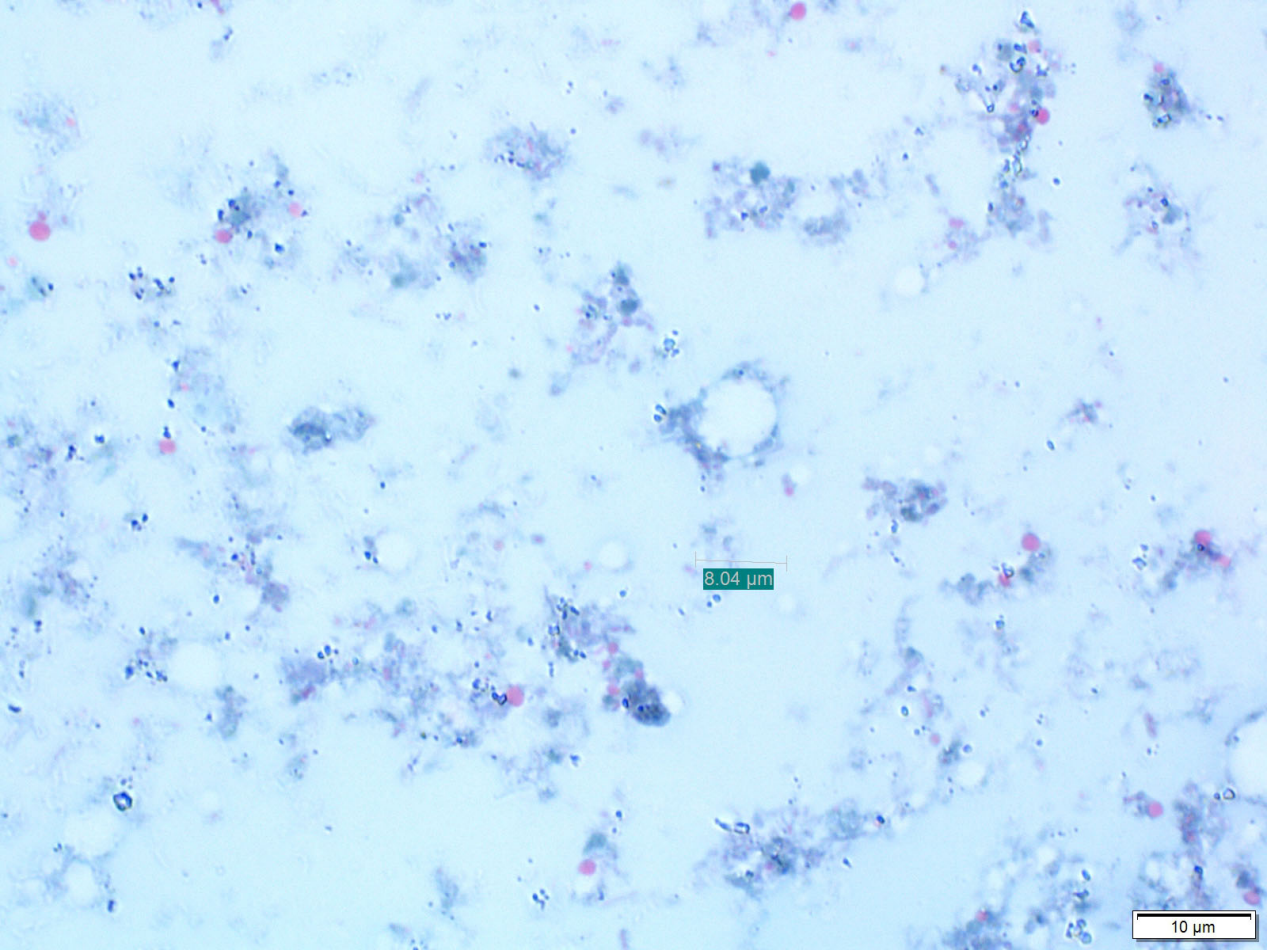
*Blastocystis* cyst (black arrow) in stool sample stained with Trichrome stain at 100x magnification objectives using light microscopy.

Stool samples macroscopic parameters had an insignificant association with *Blastocystis* infection. Thirteen samples were positive for *Blastocystis via* microscopy (**Figure 1**). Moreover, eight other protozoans and three helminths were identified in participants’ stool samples, and the majority of infections were identified in CF group. The most common protozoa found were *Entamoeba*, *Cryptosporidium*, and *Retortamonas intestinalis* (**Table S4**), and the most common helminths infection were *Enterobius vermicularis* and *Ascaris lumbricoides* (**Table S4**). *Blastocystis* co-infection was studied; however, there was no significant association with other intestinal parasites.

### 3.2 Molecular investigation: Blastocystis infection

Twenty-four samples were *Blastocystis*-positive *via* PCR and 13 *via* microscopy (**Table S5**). In total, 30 samples were *Blastocystis*-positive regardless of the detection method (**Table 1**). As anticipated, *Blastocystis* prevalence was significantly higher among all cancer types (40.4%) compared to CF group (17.3%) (*P*=0.009). Also, *Blastocystis* prevalence was significantly higher in CRC sub-group (60%) compared to CF group (*P*=0.002). However, COGT sub-group (32.4%) was insignificant compared to CF group (*P*=0.161).

**Table 1:** Logistic regression and Pearson chi-square test of *Blastocystis* infection among the study population (n = 104).

Study groups	*Blastocystis* infection				Logistic Regression Analysis		
	No. Examined	Positive n (%)	Negative n (%)	χ^2/^P-value	aOR*	95% CI	P-value
Cancer-free	52	9 (17.3)	43 (82.7)	Ref.	1	*Ref.*	–
Cancer	52	21 (40.4)	31 (59.6)	0.009	2.98	1.169-7.577	0.022
Cancer patients’ sub-groups							
COGT	37	12 (32.4)	25 (67.6)	0.161	2.28	0.825-6.291	0.112
CRC	15	9 (60)	6 (40)	0.002	5.66	1.531-20.895	0.009

P-value ≤ 0.05: indicates a statistical significance.

CI, confidence interval.

*aOR, odds ratio adjusted for age and gender, goodness of fit test: Hosmer and Lemeshow Test p>0.05. -, NA or Zero value.

The odds of *Blastocystis* infection were almost threefold higher in cancer group than in the CF group (OR=2.98, *P=*0.022) and more than fivefold higher in the CRC group (OR= 5.66, *P=*0.009). In contrast, the *Blastocystis* infection odds in COGT to CF group were insignificant (**Table 1**). *Blastocystis* fold change between CRC and COGT groups was insignificant (OR=2.35, *P=*0.209), even though the prevalence of *Blastocystis* spp. in CRC group alone was high (60%) (**Table 2**).

**Table 2:** Logistic regression and Pearson chi-square test of *Blastocystis* infection among cases, COGT vs. CRC group.

Cancer groups	*Blastocystis* infection				Logistic Regression Analysis		
	No. Examined	Positive n (%)	Negative n (%)	χ^2/^P-value	aOR*	95% CI	P-value
COGT	37	12 (32.4)	25 (67.6)	Reference	1	Reference	–
CRC	15	9 (60)	6 (40)	0.128	2.35	0.620-8.868	0.209

P-value ≤ 0.05: indicates a statistical significance.

CI, confidence interval.

*aOR: odds ratio adjusted for age and gender, goodness of fit test: Hosmer and Lemeshow Test p>0.05. -, NA or Zero value.

### 3.3 Molecular investigation: Gut fungi

Via gel electrophoresis, amplicon size of ~450-800bp using ITS primers was considered positive for gut fungi. Sixty individuals (57.7%) were tested positive for gut fungi, of which 30 were from CF group (57.7%), and 30 were from the cancer group (57.7%). Twenty-two individuals (59.5%) of the COGT subgroup had gut fungi, and 8 (53.3%) of the CRC subgroup.

Thirty-nine fungi-positive samples were sequenced. Eleven types of fungi were identified; eight were gut mycobiome, and three were environmental fungal species (**Table 3**). *Saccharomyces cerevisiae* was the most common gut fungi (n=25, 62.5%). *S. cerevisiae* was detected in 17 samples (77.3%) from CF group and 8 samples (44.4%) from the cancer group. However, *S. cerevisiae* prevalence was not significantly associated with cancer (*P*=0.193) nor *Blastocystis* spp.(*P*=0.478).

**Table 3:** Fungal species detected among the study population (n = 104).

Fungi species	Cancer-free n (%)	Cancer n (%)		Total n (%)
		COGT n (%)	CRC n (%)	
Gut fungi				
* Saccharomyces cerevisiae*	17 (77.3)	**8 (44.4)**		25 (62.5)
		7 (50)	1 (25)
* Candida glabrata*	–	**3 (16.7)**		3 (7.5)
		2 (14.3)	1 (25)
* Penicillium species*	–	**1 (5.6)**		1 (2.5)
		1 (7.1)	–
* Pichia Kudriavzevii/ Candida kruzei*	1 (4.5)	**-**		1 (2.5)
		–	–
* Aspergillus species*	–	**2 (11.1)**		2 (5)
		1 (7.1)	1 (25)
* Galactomyces geotrichum*	–	**1 (5.6)**		1 (2.5)
		1 (7.1)	–
* Candida albicans*	2 (9.1)	**1 (5.6)**		3 (7.5)
		1 (7.1)	–
* Candida tropicalis*	–	**1 (5.6)**		1 (2.5)
		1 (7.1)	–
Environmental fungi				
* Torulaspora delbruckii*	–	**1 (5.6)**		1 (2.5)
		–	1 (25)
* Hanseniaspora uvarum*	1 (4.5)	**-**		1 (2.5)
		–	–
* Kazachstania servazzii*	1 (4.5)	**-**		1 (2.5)
		–	–
Total n (%)	22 (55)	**18 (45)**		40 (100)
		14 (35)	4 (10)

Bold values indicates no.(%) of each fungus found in the cancer group.

-, NA or Zero value.

### 3.4 Blastocystis subtyping and phylogenetic analysis

Eighteen samples were *Blastocystis*-subtyped *via* sequencing. The most common *Blastocystis* subtype was ST3 (n=8, 44.4%), then ST2 (n=6, 33.3%) (**Table 4**), In our study, the most common subtype was ST3 (n=5, 55.6%) in CF group and ST2 (n=5, 55.6%) in the cancer group, and only one ST7 was detected in a breast cancer patient.

**Table 4:** Distribution of *Blastocystis* subtypes among cancer-free and cancer groups.

Study groups	Subtype 1	Subtype 2	Subtype 3	Subtype 7	Total n (%)
Cancer-free **n (%)**	3 (33.3)	1 (11.1)	5 (55.6)	0 (0)	9 (50)
Cancer **n (%)**	0 (0)	5 (55.6)	3 (33.3)	1 (11.1)	9 (50)
COGT **n (%)**	0 (0)	2 (40)	2 (40)	1 (20)	5 (27.8)
CRC **n (%)**	0 (0)	3 (75)	1 (25)	0 (0)	4 (22.2)
**Total**	**3 (16.7)**	**6 (33.3)**	**8 (44.4)**	**1 (5.6)**	**18 (100)**

Bold values indicates no.(%) of each subtype in the study population.

Fifteen *Blastocystis*-subtyped samples were assigned an accession number *via* GenBank. Allele sequence analyses showed allele 4 in all ST1-positive samples, allele 15 in three ST2-positive samples, and allele 12 in one ST2-positive sample (**Table S6**). In ST3-positive samples, allele 34 (n=2) and allele 36 (n=3) were identified. Allele 137 was identified in the ST7-positive sample.

**Figure 2:**
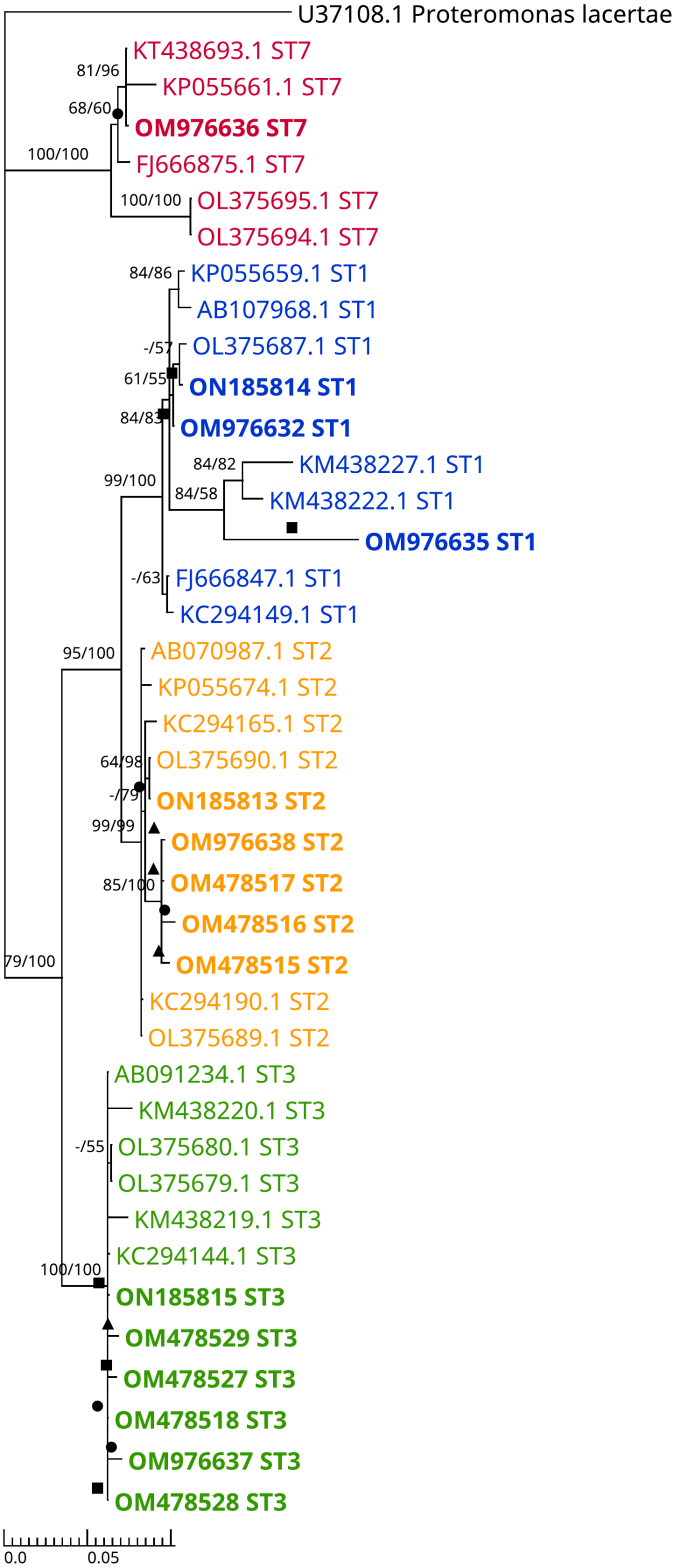
Dendrogram representing combined (ML+BI) phylogenetic tree inferred using the Barcode region of SSU rRNA gene sequences. This tree includes 15 isolated samples, 19 reference sequences from the GenBank (isolates from Cancer-free participants and CRC patients), 5 sequences of different alleles from PubMLST (AB107968.1, KT438693.1, AB070987.1, AB091234.1, and AB107965.1), and an outgroup (Proteromonas lacertae). The best tree of ML analysis was with Tamura 3-parameter model, while Bayesian tree best model was Hasegawa-Kishino-Yano model. Bootstrapping Proportions of more than 50% are shown on the left side of the branch. Bayesian posterior probability was also performed (ngen=5 million, samplefreq=100 and Burn-in 25%) and values of more than 50% are shown on the right side of the branch. A solid triangle indicates an isolate from a CRC patient, a solid circle indicates an isolate from a COGT patient and a solid square indicates an isolate from a cancer-free patient.

The ML and BI trees include 15 sample sequences submitted to NCBI GenBank with the accession numbers (OM478515-OM478518, OM478527-OM478529, OM976632, OM976635-OM976638, and ON185813-ON185815) (**Figure 2**). The topologies between the original reconstructed trees with ML (**Figure S1**) and BI (**Figure S2**) were broadly consistent. **Figure S3** shows the branch lengths of the combined tree.

## 4 Discussion

Cancer is one of the most common causes of death worldwide (15, 42). The majority of new cases in the UAE are among women compared to men. (42). Colorectal, skin, and prostate cancers are the most prevalent in men, whereas breast, thyroid, and colorectal cancers are the most common in women. (42). In our study, *Blastocystis* spp. was significantly higher in cancer patients (OR=2.98). Similarly, a regional study from the Kingdom of Saudi Arabia (KSA) reported a significant association between *Blastocystis* infection and patients with cancer (OR=2.15) (43).

CRC is among the most diagnosed malignancies and mortalities globally (15). Most incident cancers among Emirati males were related to CRC (16). In the current study, approximately 60% of CRC patients were infected with *Blastocystis*, which was higher than in previous studies in Iran (23.9%), KSA (29.7%), Egypt (52%), Turkey (7.5%), and Poland (12%) (43–47). The disparities might be attributed to the *Blastocystis* detection method or the population’s diversity (48). Consistent with the results of other studies, our study reported significantly high odds of *Blastocystis* in CRC patients (43–46).

*Blastocystis* spp. prevalence in the COGT group (32.4%) was insignificant compared to CF group (17.3%), similar to previous studies (43, 49). On the other hand, Taşova et al. reported a significant difference in *Blastocystis* prevalence between hematologic cancer patients (13%) and the non-cancer GIT patients group (1%) (50).

*Blastocystis* prevalence did not significantly differ within the cancer types included in our study (*P*=0.440). Furthermore, *Blastocystis* prevalence between CRC (60%) and COGT (32.4%) groups was insignificant (*P*=0.128), in agreement with previous studies on cancer groups (47, 51, 52). While Yersal and colleagues reported significantly higher *Blastocystis* prevalence in lung cancer (38.1%) compared to other cancer types (7.2-8.9%) (47).

In this study, patients’ medical records were assessed to identify potential risk factors associated with *Blastocystis* in cancer patients. *Blastocystis* prevalence association with risk factors tested was found insignificant (**Table S3**), as reported in other studies (44, 52).

Except for two patients (breast and hematologic/blood malignancies), all cancer patients in our study had received cancer therapy. Twenty-four cancer patients received chemotherapy, of which 18 (75%) were COGT, and 6 (25%) were CRC. The potential association of the number of completed chemotherapy cycles with *Blastocystis* prevalence was insignificant (*P*=0.705). In contrast, other studies reported that patients receiving at least eight chemotherapy cycles had a significantly higher *Blastocystis* prevalence than patients receiving fewer cycles (47, 53). Generally, *Blastocystis* infections were detected consistently in patients receiving ≥8 chemotherapy cycles compared to earlier cycles, and compared to patients who did not start treatment (47, 53, 54). Interestingly, current study findings and previous studies suggest that CRC patients had a significantly higher *Blastocystis* prevalence than healthy participants, regardless of their treatment status (43, 45, 46). Also, COGT patients receiving multiple chemotherapy cycles presented a significantly higher *Blastocystis* infection than healthy participants (43, 50). These findings may help in understanding the complex relationship between *Blastocystis* and cancer and the effects of chemotherapy on *Blastocystis* prevalence.

We have detected more *Blastocystis* infection in males (36.4%) compared to females (23.3%), in agreement with previous studies which have reported that *Blastocystis* was higher in males (11.8-53.8%) versus females (0-46.2%) (2, 44, 45, 47, 53). Ali et al. linked the previous findings to more outdoor activities and exposure to infection sources in male patients (44).

Reportedly, the gut fungi *Aspergillus flavus*, *Debaryomyces hansenii*, *Mucor mucedo*, *Mucor racemosus*, and *Issatchenkia terricola* were significantly higher in the presence of *Blastocystis* (30). Therefore, we investigated the relationship between *Blastocystis* and gut fungi in cancer patients. Fungal sequences were identified *via* nBLAST and confirmed as gut fungi *via* the human gut mycobiome database published previously (28, 36, 55, 56). However, none of the aforementioned fungi were detected in our fungi-positive samples. We have detected *S. cerevisiae* in most gut fungi-positive samples 25 (62.5%). Various studies saw *S. cerevisiae* to inhibit CRC progression and metastasis and stimulate apoptosis of cancer cells (57, 58). *S. cerevisiae* was more prevalent in CF group (77.3%) compared to cancer patients (44.4%) with an insignificant difference (*P*=0.193). The latter observation was consistent with previous studies, except the association was statistically significant (59, 60).

The predominant *Blastocystis* subtype in this study was ST3 (n=5) in CF group and ST2 (n=5) in cancer patients. In accordance with studies from Egypt and the UAE, where ST3 was the most common subtype in control participants (44, 61). On the other hand, studies in KSA and France showed ST2 and ST4 are the most common subtypes in a healthy population (43, 62). Moreover, ST2 was the most common sub-type in cancer group (n=5) and CRC patients(n=3), unlike other studies where ST3 and ST1 were the most predominant sub-type in cancer patients (43, 44, 46, 47, 51, 53).

In our study, the predominant *Blastocystis* subtypes are ST2 and ST3 in COGT group, with the same percentage (40%). Similarly, in Mohamed et al. work, *Blastocystis* ST2 was predominantly seen in COGT patients (43.7%) (43). Also, we found ST7 in one breast cancer patient while Ali et al. and Poirier et al. found ST7 in two CRC patients and one hematologic cancer patient, respectively (44, 62). Those difference in subtyping is possibly due to detection methods variation (43, 47, 63).

To our knowledge, this is the first study in the UAE to investigate *Blastocystis* intra-subtypes (alleles) variations in cancer patients. Few studies examined *Blastocystis* alleles prevalence in healthy individuals (61, 64). In our research, *Blastocystis* ST3 alleles (34 and 36) were identified in 3 cancer patients and 2 CF participants (**Table S6**). These alleles were also identified in studies conducted on healthy individuals (61, 64). We detected *Blastocystis* ST1 allele 4 in CF participants, and ST2 alleles 15 and 12 in cancer patients. While AbuOdeh et al., in the UAE, reported ST1 allele 4, and ST2 allele 9 in healthy subjects (61). We identified *Blastocystis* ST7 allele 137, a similar finding was reported in Rezaei et al. study (65). A study from Turkey reported alleles 2, 4, and 88 of ST1, and alleles 34 and 36 of ST3 in cancer patients (66).

We submitted *Blastocystis* allele sequences OM478515 ST2 and OM478527 ST3 to PubMLST and 15 of the first UAE *Blastocystis* isolates. In this study, *Blastocystis*-ST1-positive controls (n=4) were all of allele 4, ST2-positive controls (n=2) were of allele 9, and ST3-positive controls (n=2) were of allele 34 and allele 36.

In conclusion, *Blastocystis* infection was significantly associated with cancer, with ST2 being the most common subtype. Furthermore, CRC patients had a higher risk of *Blastocystis* infection than CF. Since *Blastocystis* infection is more common among cancer patients than CF individuals, further studies are needed to understand the association between *Blastocystis* infection and cancer in general, and CRC in particular. Thus, routine *Blastocystis* infection screening in cancer patients might be a useful tool to be added to the usual patient’s care in the future.

## Data availability statement

The datasets presented in this study can be found in online repositories. The names of the repository/repositories and accession number(s) can be found below: https://www.ncbi.nlm.nih.gov/genbank/, OM478529 https://www.ncbi.nlm.nih.gov/genbank/, OM478517 https://www.ncbi.nlm.nih.gov/genbank/, OM478518 https://www.ncbi.nlm.nih.gov/genbank/, OM478516 https://www.ncbi.nlm.nih.gov/genbank/, OM478528 https://www.ncbi.nlm.nih.gov/genbank/, OM478526 https://www.ncbi.nlm.nih.gov/genbank/, OM478525 https://www.ncbi.nlm.nih.gov/genbank/, OM478521 https://www.ncbi.nlm.nih.gov/genbank/, OM478519 https://www.ncbi.nlm.nih.gov/genbank/, OM478513 https://www.ncbi.nlm.nih.gov/genbank/, OM478520 https://www.ncbi.nlm.nih.gov/genbank/, OM478514 https://www.ncbi.nlm.nih.gov/genbank/, OM478512 https://www.ncbi.nlm.nih.gov/genbank/, OM478511 https://www.ncbi.nlm.nih.gov/genbank/, OM976635 https://www.ncbi.nlm.nih.gov/genbank/, OM976632 https://www.ncbi.nlm.nih.gov/genbank/, OM976639 https://www.ncbi.nlm.nih.gov/genbank/, ON185815 https://www.ncbi.nlm.nih.gov/genbank/, ON185814 https://www.ncbi.nlm.nih.gov/genbank/, OM976638 https://www.ncbi.nlm.nih.gov/genbank/, OM976637 https://www.ncbi.nlm.nih.gov/genbank/, OM976636 https://www.ncbi.nlm.nih.gov/genbank/, ON185813 https://www.ncbi.nlm.nih.gov/genbank/, OM976633 https://www.ncbi.nlm.nih.gov/genbank/, OM976634.

## Ethics statement

The studies involving human participants were reviewed and approved by Tawam Human Research Ethics Committee (T-HREC) of Tawam Hospital, Al Ain, Abu Dhabi, UAE (THREC-678). The patients/participants provided their written informed consent to participate in this study.

## Author contributions

LL: Methodology, data curation, analysis, interpretation, visualization, writing, and editing. SZ: Conception and design, methodology, data analysis, interpretation, revision, and writing. SA: Methodology, interpretation, writing, revision, and editing. MO: Methodology, analysis, and revision. SS: Study design and methodology. ZR: Conception and design, supervision, funding acquisition, methodology, data analysis, interpretation, revision, and editing. All authors contributed to the article and approved the submitted version.
