# Supplementary material for: The associated risk of Blastocystis infection in cancer: A case control study
Source: Front Oncol. 2023 Feb 20;13:1115835. doi: 10.3389/fonc.2023.1115835 (PMC9986623; doi:10.3389/fonc.2023.1115835)
Supplement: Supplementary file 1 [file DataSheet_1.docx]

| **Table S1**: List of Primers and PCR Conditions used in this study | | | | | |
| --- | --- | --- | --- | --- | --- |
| **Target gene** | **Primers** | **Sequences** | **PCR Condition** | **Expected Product Size** | **Reference** |
| Barcode region  (SSU rRNA gene) *Blastocystis* | **RD5** | 5′-ATC TGG TTG ATC CTG CCA GT-3′ | 94°C for 5 min.,  **35x** (94°C for 30 sec  55°C for 30 sec.  72°C for 1 min.)  72°C for 7 min. | ~600bp | https://doi.org/10.1128/AEM.00520-15 |
|  | **BhRDr** | 5′-GAG CTT TTT AAC TGC AAC AAC G-3′ |  |  |  |
| Barcode region  (ITS regions)  Gut fungi | **ITS1F** | 5′-CTT GGT CAT TTA GAG GAA GTA A-3′ | 95° for 5 min.,  **35x** (94°C for 30 sec.  56°C for 30 sec.  72°C for 1 min.)  72°C for 8 min. | ~450-800 bp | <https://doi.org/10.1021/acs.jnatprod.6b01085> |
|  | **ITS4** | 5′-TCC TCC GCT TAT TGA TAT GC-3′ |  |  |  |

| **Table S2**: Characteristics of the study population | | | |
| --- | --- | --- | --- |
| **Variables** | | **(n)** | **(%)*** |
| **Gender** |  | | |
|  | Males | 44 | 42.3 |
|  | Females | 60 | 57.7 |
| **Age mean, median (range)** 46.3, 48, (22-87) | | | |
|  | Youth (18-24 yrs.) | 6 | 5.8 |
|  | Adults (25-59 yrs.) | 84 | 80.8 |
|  | Elderly (>60 yrs.) | 14 | 13.5 |
| **Nationality** |  | | |
|  | Emirati | 29 | 27.9 |
|  | Syrian | 9 | 8.7 |
|  | British | 1 | 1 |
|  | Indian | 8 | 7.7 |
|  | Egyptian | 8 | 7.7 |
|  | Lebanese | 1 | 1 |
|  | Palestinian | 3 | 2.9 |
|  | Filipino | 8 | 7.7 |
|  | Sudanese | 3 | 2.9 |
|  | Omani | 4 | 3.8 |
|  | Ethiopian | 3 | 2.9 |
|  | Yemeni | 3 | 2.9 |
|  | Bangladeshi | 9 | 8.7 |
|  | Nepali | 1 | 1 |
|  | Somali | 3 | 2.9 |
|  | Eritrean | 1 | 1 |
|  | Iraqi | 1 | 1 |
|  | Jordanian | 4 | 3.8 |
|  | Canadian | 1 | 1 |
|  | Pakistani | 3 | 2.9 |
|  | South African | 1 | 1 |
| **Regions** |  | | |
|  | Africa | 5 | 4.8 |
|  | Americas | 1 | 1 |
|  | South-East Asia | 18 | 17.3 |
|  | Europe | 1 | 1 |
|  | Eastern Mediterranean | 71 | 68.3 |
|  | Western Pacific | 8 | 7.7 |
| **Cancer-free participants** | | **52** | **50** |
| **Cancer patients** | | **52** | **50** |
|  | CRC | 15 | 14.4 |
|  | COGT | 37 | 35.6 |
|  | *Breast* | 16 | 15.4 |
|  | *Hematologic* | 9 | 8.7 |
|  | *Gynecologic* | 3 | 2.9 |
|  | *Accessory* | 3 | 2.9 |
|  | *Lung* | 2 | 1.9 |
|  | *Genitourinary* | 2 | 1.9 |
|  | *Germ Cell* | 1 | 0.96 |
|  | *Head and Neck* | 1 | 0.96 |
| **Total** |  | **104** | **100** |
| *Percentages were calculated out of 104 | | | |

| **Table S3**: Association between cancer treatment with *Blastocystis* infection among the cancer group | | | | | |
| --- | --- | --- | --- | --- | --- |
| **Variables** |  | ***Blastocystis*** | | **P-value** | **Total**  **n (%)** |
|  |  | **Positive**  **n (%)** | **Negative**  **n (%)** |  |  |
| **Cancer treatment** | | | | |  |
|  | Chemotherapy | 11 (45.8) | 13 (54.2) | 0.341 | 24 (48) |
|  | Immunotherapy | 4 (57.1) | 3 (42.9) |  | 7 (14) |
|  | Radiotherapy | 1 (100) | - |  | 1 (2) |
|  | Chemotherapy + Immunotherapy | 4 (25) | 12 (75) |  | 16 (32) |
|  | Radiotherapy + Chemotherapy | - | 1 (100) |  | 1 (2) |
|  | Chemotherapy + Radiotherapy + Immunotherapy | - | 1 (100) |  | 1 (2) |
| **Total** | | **20 (40)** | **30 (60)** |  | **50 (100)*** |
| **Chemotherapy Cycles** | | | | | |
|  | Cycle 1 | 3 (50) | 3 (50) | 0.705 | 6 (25) |
|  | Cycle 2 | - | 2 (100) |  | 2 (8.3) |
|  | Cycle 3 | - | 1 (100) |  | 1 (4.2) |
|  | Cycle 4 | 2 (40) | 3 (60) |  | 5 (20.8) |
|  | Cycle 5 | 1 (33.3) | 2 (66.7) |  | 3 (12.5) |
|  | Cycle 7 | 1 (100) | - |  | 1 (4.2) |
|  | Cycle 8 | 2 (100) | - |  | 2 (8.3) |
|  | Cycle 12 | 2 (50) | 2 (50) |  | 4 (16.7) |
| **Total** | | **11 (45.8)** | **13 (54.2)** |  | **24 (100)**** |
| P-value ≤ 0.05: indicates a statistical significance  *Percentages were calculated out of 50 who received treatment (2 did not receive any treatment) at the time of sample collection  **Percentages were calculated out of 24 who received only chemotherapy | | | | | |

| **Table S3:** Association between clinical data with *Blastocystis* infection among the cancer group (Continued) | | | | | |
| --- | --- | --- | --- | --- | --- |
| **Variables** | | ***Blastocystis spp.*** | | **P value** | **Total**  **n (%)** |
|  |  | **Positive**  **n (%)** | **Negative**  **n (%)** |  |  |
| **Antibiotics** | | | | |  |
|  | Yes | 3 (30) | 7 (70) | 0.721 | 10 (19.2) |
|  | No | 18 (42.9) | 24 (57.1) |  | 42 (80.8) |
| **Hospitalization status** | | | | |  |
|  | Inpatient | 3 (18.8) | 13 (81.3) | 0.07 | 16 (30.8) |
|  | Outpatient | 18 (50) | 18 (50) |  | 36 (69.2) |
| **Total** | | **21 (40.4)** | **31 (59.6)** |  | **52 (100)*** |
| **Surgical procedures history** | | | | | |
|  | Yes | 9 (56.3) | 7 (43.8) | 0.241 | 16 (31.4) |
|  | No | 12 (34.3) | 23 (65.7) |  | 35 (68.6) |
| **Total** | | **21 (42.2)** | **30 (58.8)** |  | **51 (100)**** |
| P-value ≤ 0.05: indicates a statistical significance  *Percentages were calculated out of 52 (All cases)  **Percentages were calculated out of 51 who reported undergoing GIT surgical procedures in the last 2 years | | | | | |

| **Table S4:** Numbers and proportions of other intestinal parasites seen across study groups | | | | | |
| --- | --- | --- | --- | --- | --- |
| **Other Intestinal Parasites** | | **Cancer-free n (%)** | **Cancer n (%)** | | **Total n (%)** |
|  |  |  | **COGT**  **n (%)** | **CRC**  **n (%)** |  |
| **Intestinal Protozoa** | | | | | |
|  | *Entamoeba spp.* | 3 (20) | - | | 3 (13) |
|  |  |  | - | - |  |
|  | *Cryptosporidium spp.* | 3 (20) | - | | 3 (13) |
|  |  |  | - | - |  |
|  | *Cyclospora cayetanensis* | - | **1 (12.5)** | | 1 (4.3) |
|  |  |  | 1 (16.7) | - |  |
|  | *Entamoeba coli* | 1 (6.7) | **1 (12.5)** | | 2 (8.7) |
|  |  |  | 1 (16.7) | - |  |
|  | *Entamoeba hartmanni* | 1 (6.7) | **1 (12.5)** | | 2 (8.7) |
|  |  |  | 1 (16.7) | - |  |
|  | *Chilomastix mesnili* | - | **1 (12.5)** | | 1 (4.3) |
|  |  |  | 1 (16.7) | - |  |
|  | *Microsporidia* | - | **1 (12.5)** | | 1 (4.3) |
|  |  |  | - | 1 (50) |  |
|  | *Retortamonas intestinalis* | - | **3 (37.5)** | | 3 (13) |
|  |  |  | 2 (33.3) | 1 (50) |  |
| **Intestinal Helminths** |  |  |  |  |  |
|  |  |  |  |  |  |
|  | *Enterobius vermicularis* | 3 (20) | - | | 3 (13) |
|  |  |  | - | - |  |
|  | *Ascaris lumbricoides* | 3 (20) | - | | 3 (13) |
|  |  |  | - | - |  |
|  | *Trichuris* *trichiura* | 1 (6.7) | - | | 1 (4.3) |
|  |  |  | - | - |  |
| Total n (%) | | 15 (28.8) | **8 (34.8)** | | 23 (100) |
|  |  |  | 6 (26.1) | 2 (8.7) |  |

| **Table S5**: Detection of *Blastocystis* using different diagnostic methods among the study population (n = 104) | | | |
| --- | --- | --- | --- |
| **PCR** | **Light Microscopy** | | |
|  | **Positive n (%)** | **Negative n (%)** | **Total n (%)*** |
| Positive n (%) | 7 (6.7) | 17 (16.3) | **24 (23.1)** |
| Negative n (%) | 6 (5.8) | 74 (71.2) | **80 (76.9)** |
| **Total n (%)*** | **13 (12.5)** | **91 (87.5)** | **104 (100)** |
| *Percentages were calculated out of 104 | | | |

| **Table S6:** Distribution of alleles of *Blastocystis* subtypes among the study population (n=104) | | |
| --- | --- | --- |
| **Allele** | **Subtype** | **Group** |
| 4 | 1 | CF |
| 4 | 1 | CF |
| 4 | 1 | CF |
| 12 | 2 | Breast |
| 15 | 2 | CRC |
| 15 | 2 | CRC |
| 15 | 2 | Lung |
| 34 | 3 | CRC |
| 34 | 3 | CF |
| 36 | 3 | CF |
| 36 | 3 | Hematologic |
| 36 | 3 | Hematologic |
| 137 | 7 | Breast |
| 155* | 2 | CRC |
| 156* | 3 | CF |
| *Newly discovered *Blastocystis* alleles in this study and submitted to PubMLST | | |


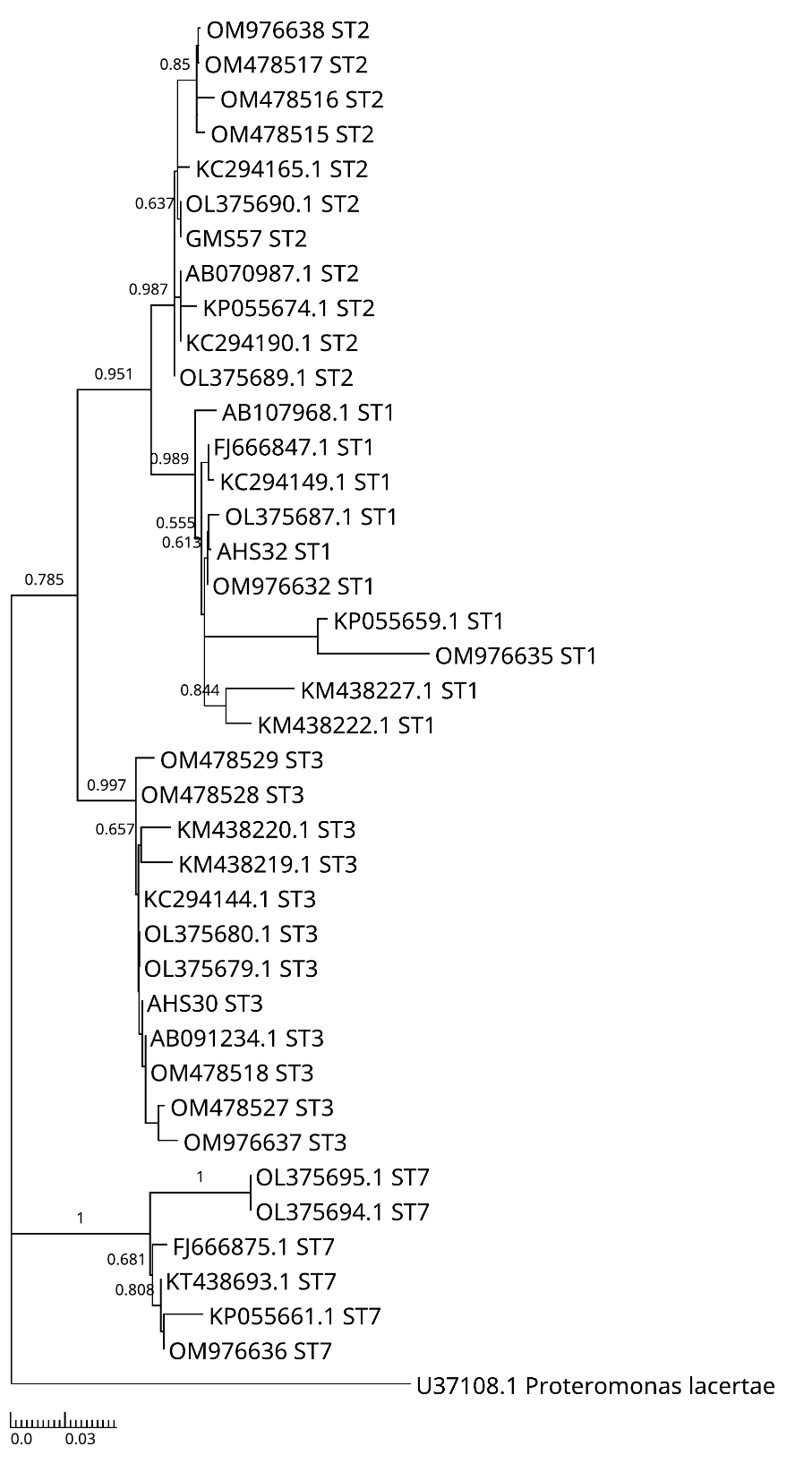


**Figure S1:** Dendrogram representing Maximum Likelihood (ML) phylogenetic tree of *Blastocystis* identified subtypes (STs) from the Cancer-free and cancer groups. Bootstrapping Proportions of more than 0.5 are shown on the branch





**Figure S2:** Dendrogram representing Bayesian Inference (BI) phylogenetic tree of *Blastocystis* identified subtypes (STs) from the Cancer-free and cancer groups. Bayesian posterior probability of more than 50% are shown on the branch.


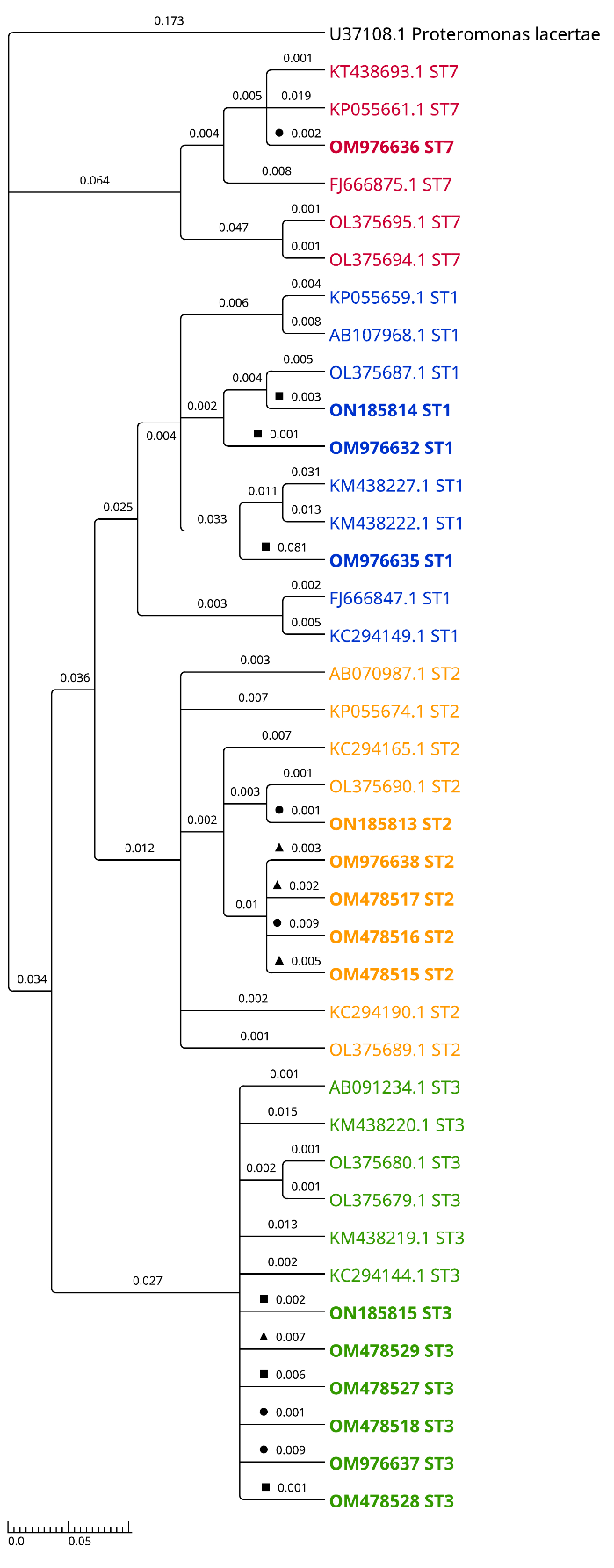


**Figure S3:** Dendrogram representing combined (ML+BI) phylogenetic tree of *Blastocystis* identified subtypes (STs) from the Cancer-free and cancer groups. Branch length shown on the top of each branch. A solid triangle indicates an isolate from a CRC patient, a solid circle indicates an isolate from a COGT patient, and a solid square indicates an isolate from a cancer-free patient.
